# Pembrolizumab monotherapy versus pembrolizumab plus chemotherapy in patients with non‐small‐cell lung cancer: A multicenter retrospective trial

**DOI:** 10.1111/1759-7714.14252

**Published:** 2021-12-05

**Authors:** Hiromi Matsumoto, Nobuaki Kobayashi, Kohei Somekawa, Nobuhiko Fukuda, Ayami Kaneko, Chisato Kamimaki, Sousuke Kubo, Katsushi Tanaka, Yoichi Tagami, Shuhei Teranishi, Keisuke Watanabe, Nobuyuki Horita, Yu Hara, Masaki Yamamoto, Makoto Kudo, Harumi Koizumi, Kenji Miura, Naoki Miyazawa, Takeshi Kaneko

**Affiliations:** ^1^ Department of Pulmonology Yokohama City University Graduate School of Medicine Yokohama Japan; ^2^ Respiratory Disease Center Yokohama City University Medical Center Yokohama Japan; ^3^ Department of Pulmonology Yokohama Minami Kyosai Hospital Yokohama Japan; ^4^ Respiratory Disease Center Yokohama Sakae Kyosai Hospital Yokohama Japan; ^5^ Department of Pulmonology Saiseikai Yokohamashi Nanbu Hospital Yokohama Japan

**Keywords:** metastasis, non‐small‐cell lung cancer, pembrolizumab

## Abstract

**Background:**

Pembrolizumab alone or in combination with chemotherapy is a standard treatment for patients with non‐small‐cell lung cancer (NSCLC) with high programmed death‐ligand 1 (PD‐L1) expression. However, no study has compared the efficacies of these two regimens. Therefore, we aimed to compare the efficacy of pembrolizumab alone and in combination with chemotherapy in NSCLC patients with high PD‐L1 expression.

**Methods:**

We conducted a multicenter retrospective trial involving patients with diagnosed unresectable or recurrent NSCLCs who had received pembrolizumab with or without chemotherapy in the first‐line setting. Patients were divided into monotherapy and combination therapy groups. The progression‐free survival (PFS), overall survival (OS), and response rate (RR) were analyzed and compared between the groups. Clinical characteristics of patients were analyzed to assess their possible relationship with treatment outcomes.

**Results:**

We enrolled 96 patients from five hospitals. Of these, 47 and 49 patients received monotherapy and combination therapy, respectively. The median PFS was 343 and 328 days in the monotherapy and combination therapy groups, respectively (hazard ratio 1.003, *p* = 0.99). No statistically significant differences were observed in the OS and RR between the two groups. However, in patients with metastases to the liver, lung, adrenal glands, bone, or lymph nodes, the PFS was longer in the monotherapy group than in the combination therapy group.

**Conclusion:**

Although the PFS, OS, and RR were not significantly different between patients treated with pembrolizumab alone and or with pembrolizumab in combination with chemotherapy, patients with NSCLC having metastases to specific sites may benefit more from monotherapy.

## Graphical abstract

We conducted a multicenter retrospective trial involving patients with diagnosed unresectable or recurrent non‐small‐cell lung cancer who had received pembrolizumab with or without chemotherapy in the first‐line setting. The median progression‐free survival (PFS) was 343 and 328 days in the monotherapy and combination therapy groups, respectively (a). In patients with metastases to the liver, lung, adrenal glands, bone, or lymph nodes, the PFS was longer in the monotherapy group than in the combination therapy group (b).

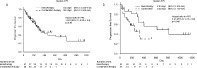

## INTRODUCTION

Lung cancer is the leading cause of cancer‐related deaths worldwide. Most patients with lung cancer are diagnosed when the disease has progressed to an advanced stage, therefore there is a need to develop better treatment options, especially for patients diagnosed with lung cancer at an advanced stage.^1^, ^2^ Traditional standard treatment for patients with lung cancer without oncogenic driver mutations is chemotherapy with cytotoxic agents; however, their effectiveness is very limited. Recently, immune checkpoint inhibitors (ICIs) have changed the treatment paradigm for these patients.^3^

Pembrolizumab, an antiprogrammed cell death protein 1 (PD‐1) antibody, was approved for the treatment of advanced non‐small‐cell lung cancer (NSCLC), following the approval of nivolumab. In 2016, pembrolizumab was shown to lead to a superior progression‐free survival (PFS) and overall survival (OS) compared to conventional chemotherapy in patients with lung cancer who had a high expression of programmed cell death‐ligand 1 (PD‐L1).^4^ Based on the results of clinical trials, monotherapy with pembrolizumab has become one of the standard treatment options for patients with advanced NSCLC whose tumors show a high PD‐L1 expression.^5^, ^6^ In 2018, the combination of pembrolizumab with chemotherapy was shown to be superior to chemotherapy alone, regardless of the PD‐L1 expression level, in phase‐III trials.^7^, ^8^ Based on these results, the National Comprehensive Cancer Network (NCCN) guidelines recommend pembrolizumab monotherapy or pembrolizumab in combination with chemotherapy for patients with NSCLC who have a high PD‐L1 tumor proportion score (TPS).^6^

However, none of the clinical trials have compared the efficacies of pembrolizumab monotherapy and pembrolizumab in combination with chemotherapy in patients with NSCLC who have a high PD‐L1 expression. Atezolizumab, another ICI, is in a similar situation.^9^, ^10^, ^11^ ^12^

Therefore, in the present study, we compared the efficacy of pembrolizumab alone and in combination with chemotherapy in patients with NSCLC who had a high PDL‐1 expression, using real‐world data collected from multiple centers.

## METHODS

### Patients

In this study, we retrospectively analyzed the data from patients with NSCLC treated at the Yokohama City University Medical Center, Yokohama Minami Kyosai Hospital, Yokohama Sakae Kyosai Hospital, Saiseikai Yokohamashi Nanbu Hospital, and Yokohama City University Hospital between January 2016 and August 2019. Patients with pathologically diagnosed unresectable or recurrent NSCLC who had received treatment either with pembrolizumab alone or pembrolizumab in combination with chemotherapy in the first‐line setting were included in the study. Patients who had received adjuvant chemotherapy prior to first‐line treatment were also included. Patients who discontinued treatment during the first course of treatment or whose data records were incomplete were excluded from this study. Data related to the patients' characteristics, pathological type, PD‐L1 TPS, PFS, OS, and adverse events were obtained from the medical records. The study protocol was approved by our institutional review board (approval number: B191200044), and the need to obtain written informed consent from the patients was waived due to the retrospective nature of the study.

### Treatments

The monotherapy group included patients who were treated with pembrolizumab alone, while the combination therapy group included patients who were treated with a combination of pembrolizumab and platinum‐doublet chemotherapy. All the patients included in the combination therapy group had received one of the following two platinum‐doublet chemotherapy regimens: carboplatin/cisplatin with pemetrexed or carboplatin with nanoparticle albumin‐bound [nab]‐paclitaxel/paclitaxel.

### Endpoints

The primary endpoints of the study were PFS, OS, and response rate (RR) after treatment initiation. The secondary endpoint was adverse events due to chemotherapy.

PFS was defined as the time between the date of treatment initiation and the date of confirmation of progressive disease. OS was defined as the time between the date of treatment initiation and date of death.

Response was assessed using the Response Evaluation Criteria in Solid Tumors (RECIST) version 1.1. Adverse events and abnormal laboratory findings were graded according to the National Cancer Institute Common Terminology Criteria for Adverse Events, version 5.0.

### Statistical analysis

Data were analyzed using JMP Pro 15 software (SAS Institute Inc.). The chi‐squared test and Mann–Whitney U test were used to assess the differences between the monotherapy and combination therapy groups. The Kaplan–Meier method was used to estimate the PFS and OS. The log‐rank test was used to assess the difference in survival between the two groups. Hazard ratios (HRs) were calculated using the Cox proportional hazards model. A *p* value of less than 0.05 was considered statistically significant.

## RESULTS

### Patient characteristics

A total of 96 patients from five hospitals were enrolled in this study (Table 1). Of these, 47 patients received pembrolizumab monotherapy and 49 received pembrolizumab in combination with chemotherapy. The median age of the patients was 71 years (range 36–87 years) and 68 years (range 52–81 years) in the monotherapy and combination therapy groups, respectively (*p* = 0.0113).

**TABLE 1:** Patient characteristics and demographics at baseline

	Monotherapy	Combination therapy	*p* value
Number of patients	47	49	
Age (range)	71 (36–87)	68 (52–81)	0.011
Sex			
Male	37	41	0.53
Female	10	8	
ECOG PS (%)			
0	22 (46.8%)	27 (55.1%)	0.42
1	25 (53.2%)	22 (44.9%)	
Smoking status (%)			
Current/former	39 (83.0%)	42 (85.7%)	0.37
Never	8 (17.0%)	5 (10.2%)	
Unknown	0 (0.0%)	2 (4.1%)	
Pathology (%)			
Nonsquamous	29 (61.7%)	37 (75.5%)	0.14
Squamous	18 (38.3%)	12 (24.5%)	
PD‐L1 TPS (%)			
<1%	0 (0.0%)	10 (20.4%)	<0.0001
1–49%	1 (2.1%)	15 (20.4%)	
≧50%	46 (97.9%)	20 (40.8%)	
Unknown	0 (0.0%)	4 (8.2%)	
Driver mutation (%)			
Positive	0 (0.0%)	0 (0.0%)	
Negative	44 (93.6%)	46 (93.9%)	
Unknown	3 (6.4%)	3 (6.1%)	
Stage (%)			
IIIB	3 (6.4%)	1 (2.0%)	
IIIC	2 (4.3%)	1 (2.0%)	
IVA	25 (53.2%)	21 (42.9%)	
IVB	6 (12.8%)	18 (36.7%)	
Recurrence after surgery	11 (23.4%)	8 (16.3%)	
NLR (range)	2.64 (1.20–29.0)	3.53 (1.03–14.51)	0.38

Abbreviations: ECOG PS, Eastern Cooperative Oncology Group performance status; NLR, neutrophil‐to‐lymphocyte ratio; PD‐L1, programmed death‐ligand 1; TPS, tumor proportion score.

In the monotherapy group, 22 and 18 patients had non‐squamous cell carcinoma and squamous cell carcinoma, respectively. In the combination therapy group, 35 and 12 patients had non‐squamous cell carcinoma and squamous cell carcinoma, respectively.

A comparison of patient characteristics between the monotherapy and combination therapy groups revealed that the number of patients with a high PD‐L1 expression (TPS ≥ 50%) was significantly greater in the monotherapy group than in the combination therapy group (*p* < 0.0001). However, there were no significant differences in sex, Eastern Cooperative Oncology Group performance status (ECOG PS), driver mutation status, clinical stage, or smoking history between the two groups. The median duration of follow‐up in the monotherapy group was 379 (range 58–1169) days and 271 (range 44–552) days in the combination therapy group; this difference was statistically significant (*p* = 0.0018).

### Efficacy

Progression‐free survival (PFS) and overall survival (OS).

**FIGURE 1:**
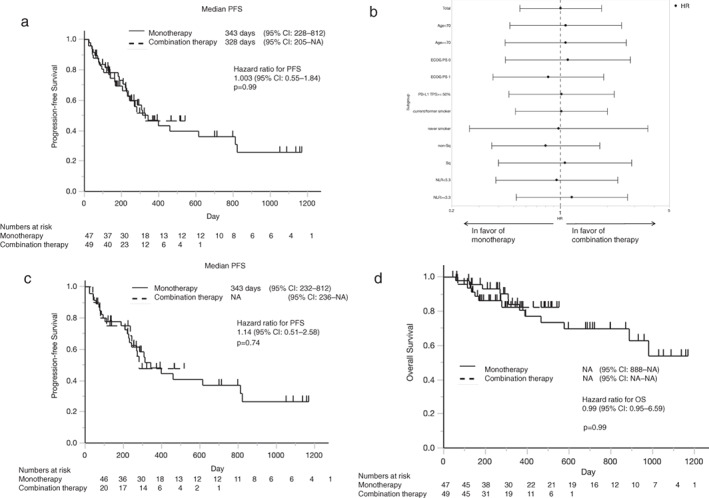
(a) Kaplan–Meier analysis of progression‐free survival in the monotherapy and combination therapy groups. The tick marks indicate the cases that were confirmed to be alive or censored at the end of the observation period. There was no significant difference between the two groups (*p* = 0.99, HR 1.003). (b) Hazard ratio for progression‐free survival by subgroup analysis, based on patient characteristics; ranges represent 95% confidence interval. There were no significant differences between the subgroups. (c) Kaplan–Meier analysis of progression‐free survival in the monotherapy and combination therapy groups for patients with a high programmed death‐ligand 1 expression. (d) Kaplan–Meier analysis of overall survival in the monotherapy and combination therapy groups; there was no significant difference between the two groups (*p* = 0.99, HR 0.99). CI, confidence interval; ECOG PS, Eastern Cooperative Oncology Group performance status; HR, hazard ratio; non‐sq, nonsquamous cell carcinoma; NRL, neutrophil‐to‐lymphocyte ratio; OS, overall survival PD‐L1, programmed cell death‐ligand 1; PFS, progression‐free survival; sq, squamous cell carcinoma; TPS, tumor proportion score

The median PFS was 343 days in the monotherapy group and 328 days in the combination therapy group (log‐rank *p* = 0.99, HR 1.003, 95% confidence interval [CI] 0.54–1.85) (Figure 1a). A subgroup analysis of PFS by age, ECOG PS, PD‐L1 TPS, histological type, smoking history, brain metastasis, and neutrophil‐to‐lymphocyte ratio (NLR) was performed, but no significant differences were observed (Figure 1b). Among the patients with a high PD‐L1 expression (TPS ≥ 50%), the median PFS was 343 days in the monotherapy group (*n* = 46) and not reached in the combination therapy group (*n* = 20) (log‐rank *p* = 0.74, HR 1.14, 95% CI 0.51–2.58) (Figure 1c). The median OS was also compared between the two groups. As with PFS, there was no significant difference in the OS between the two groups (Figure 1d). The median OS was not reached in both groups (*p* = 0.99, HR 0.99, 95% CI 0.36–2.69). There was no statistical difference in OS between the two groups, even in patients limited in high PD‐L1 expression (TPS ≥ 50%) (data not shown).

### Response rate

The RR after chemotherapy was compared between the monotherapy and combination therapy groups. Complete response was observed in two patients in each group (Table 2). Similarly, partial response, stable disease, and progressive disease were observed in 24, 10, and one patient(s), respectively, in the monotherapy group, and 30, 10, and seven patients, respectively, in the combination therapy group. The objective response rate was 55.3% in the monotherapy group and 65.3% in the combination therapy group, but the difference was not statistically significant (*p* = 0.3172).

**TABLE 2:** Summary of treatment response and objective response rate

Best response	Monotherapy	Combination therapy	*p* value
CR (%)	2 (4.3%)	2 (4.1%)	
PR (%)	24 (51.1%)	30 (61.2%)	
SD (%)	10 (21.3%)	10 (20.4%)	
PD (%)	10 (21.3%)	7 (14.3%)	
Unknown	1 (2.1%)	0 (0.0%)	
ORR	55.30%	65.30%	0.63

Abbreviations: CR, complete response; SD, stable disease; ORR, objective response rate; PD, progressive disease; PR, partial response.

### Effect of metastasis on treatment outcomes

**FIGURE 2:**
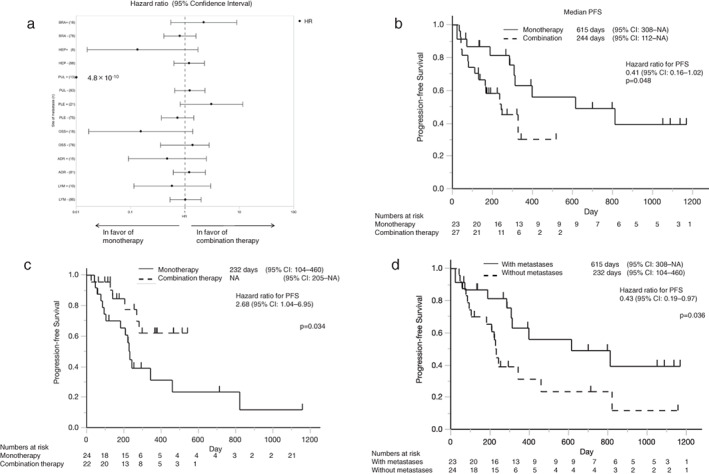
(a) Hazard ratio for progression‐free survival by subgroup analysis, based on the presence or absence of metastases to the brain, liver, lung, pleura, bone, adrenal gland, and extrathoracic lymph nodes. The progression‐free survival was longer with monotherapy than with combination therapy in the presence of liver, lung, bone, adrenal gland, and lymph node metastases. (b) Kaplan–Meier analysis of progression‐free survival in patients with metastases to the liver, lung, bone, adrenal gland, or lymph node. The progression‐free survival was significantly longer with monotherapy than with combination therapy in the presence of metastases (*p* = 0.048, HR 0.41). (c) Kaplan–Meier analysis of progression‐free survival in patients with no metastases to the liver, lung, bone, adrenal gland, or lymph nodes. The progression‐free survival of the combination therapy group was significantly better than that of the monotherapy group (*p* = 0.034, HR 2.68). (d) Kaplan–Meier analysis of progression‐free survival with and without metastasis to the liver, lung, bone, adrenal gland, or lymph nodes in the monotherapy group. Patients with metastasis to any of these sites had a longer progression‐free survival than those who did not (*p* = 0.036, HR 0.43). BRA, brain metastasis; HEP, liver metastasis; PUL, lung metastasis; PLE, pleural metastasis; OSS, bone metastasis; ADR, adrenal metastasis; LYM, lymph node metastasis; PFS, progression‐free survival; CI, confidence interval

The hazard ratios for PFS were analyzed separately for patients with metastases of the brain, liver, lung, pleura, adrenal gland, bone, and extrathoracic lymph node (Figure 2a). Although there were no statistically significant differences in the PFS based on the metastatic site, the PFS of patients who received pembrolizumab monotherapy was found to be longer than that of those who received combination therapy in the presence of metastases to the liver, lungs, adrenal glands, bone, or lymph nodes. Additionally, for patients with metastasis to at least one of the five above‐mentioned sites, the PFS of the monotherapy group was significantly longer than that of the combination therapy group (*p* = 0.048, HR 0.41, 95% CI 0.16–1.02) (Figure 2b). Contrarily, for patients without any metastasis, the PFS of the combination therapy group was longer than that of the monotherapy group (*p* = 0.034, HR 2.68, 95% CI 1.04–6.95) (Figure 2c). As metastasis to at least one of the five sites was a prognostic factor for more favorable PFS among patients treated with pembrolizumab monotherapy (Figure 2b,c), we compared the PFS of patients in the monotherapy group, with and without metastases (Figure 2d). PFS was found to be significantly longer in the presence of metastases among patients who received monotherapy (*p* = 0.036, HR 0.43, 95% CI 0.194–0.968). For patients who received combination therapy, there was no significant difference in the PFS between those with and without metastases (Supporting Information Figure S1). In the monotherapy group, the objective response rate in patients with metastases was significantly higher than that of patients without metastases (82% vs. 29%, *p* = 0.0002) (Table 3). There were no significant differences in patient characteristics between the monotherapy and combination therapy groups, except for the male/female ratio.

**TABLE 3:** Summary of treatment response and objective response rate for the monotherapy group

Best response	With metastasis	Without metastasis	*p* value
CR (%)	2 (8.7%)	0 (0.0%)	
PR (%)	17 (73.9%)	7 (29.2%)	
SD (%)	1 (4.3%)	9 (37.5%)	
PD (%)	3 (13.0%)	7 (29.2%)	
Unknown	0 (0.0%)	1 (4.2%)	
ORR	82.60%	29.20%	0.0002

Abbreviations: CR, complete response; SD, stable disease; ORR, objective response rate; PD, progressive disease; PR, partial response.

### Safety

Treatment‐related adverse events are summarized in Table 4. For patients in the monotherapy group, the median duration of treatment with pembrolizumab was 6.8 months (range 0.7–40.0 months, median number of doses 9, range 1–56); for those in the combination therapy group, the median duration of treatment with pembrolizumab and chemotherapy was 5.0 months (range 0.7–18.1 months, median number of doses 7, range 2–26). Eight and five patients discontinued treatment due to severe adverse events in the monotherapy and combination therapy groups, respectively (Table 4).

**TABLE 4:** Treatment‐related adverse events

	Monotherapy group (*n* = 47)		Combination therapy group (*n* = 49)	
	Any grade	Grade 3/4	Any grade	Grade 3/4
Any events	30 (63.8%)	4 (8.5%)	47 (95.9%)	18 (36.7%)
Discontinuation due to AE	8 (22.9%)	2 (5.7%)	5 (19.2%)	0
Neutropenia	0	0	24 (50.0%)	12 (24.5%)
Thrombocytopenia	0	0	4 (8.2%)	1 (2.0%)
Anemia	0	0	3 (6.1%)	0
AST/ALT increased	0	0	6 (12.2%)	1 (2.0%)
GGT increased	0	0	1 (2.0%)	1 (2.0%)
Fever	2 (4.3%)	0	1 (2.0%)	0
Pneumonitis	11 (23.4%)	1 (2.1%)	5 (10.2%)	0
Hematuria	1 (2.1%)	0	0	0
Arthralgia	1(2.1%)	0	0	0
Dysesthesia	1 (2.1%)	1 (2.1%)	3 (6.1%)	1(2.0%)
Hyperthyroidism	3 (6.4%)	0	0	0
Hypothyroidism	3 (6.4%)	0	4 (8.2%)	0
Anorexia	3 (6.4%)	0	4 (8.2%)	2 (4.1%)
Adrenal insufficiency	2 (4.3%)	0	3 (6.1%)	1 (2.0%)
Renal disorder	0	0	3 (6.1%)	0
Hyperkalemia	0	0	2 (6.1%)	0
Malaise	2 (4.3%)	0	3 (6.1%)	0
Skin rash	7 (14.9%)	0	7 (14.3%)	1 (2.0%)
Nausea/vomiting	1 (2.1%)	0	9 (18.4%)	2 (4.1%)
Diarrhea	0	0	7 (14.3%)	1 (2.0%)
Glucose intolerance	1 (2.1%)	0	1 (2.0%)	0
Oropharyngeal pain	1 (2.1%)	0	0	0
Myositis	1 (2.1%)	0	0	0
Colitis	1 (2.1%)	1 (2.1%)	1 (2.0%)	1 (2.0%)
Peritoneal infection	1 (2.1%)	1 (2.1%)	0	0
Abdominal pain	0	0	1 (2.0%)	0
Alopecia	0	0	1 (2.0%)	0
Hiccups	0	0	2 (4.1%)	0
Anaphylaxis	0	0	1 (2.0%)	1 (2.0%)

Abbreviations: AE, adverse events; ALT, alanine aminotransferase; AST, aspartate aminotransferase; GGT, gamma‐glutamyl transferase.

Treatment‐related adverse events were more frequent in the combination therapy group (95.9%) than in the monotherapy group (63.8%). The commonly reported adverse events (>10%) in the monotherapy group included pneumonitis and skin rash, whereas those in the combination therapy group included neutropenia, nausea, vomiting, increased alanine aminotransferase and aspartate aminotransferase levels, diarrhea, pneumonitis, and skin rash (Table 4).

## DISCUSSION

In this retrospective study, we compared the effectiveness and safety of pembrolizumab alone and in combination with chemotherapy in patients with advanced NSCLC. We observed that the PFS and OS were similar in the monotherapy and combination therapy groups, but adverse events were less frequent in the monotherapy group (Figures 1a–d and Tables 1, 2, 3, 4). Interestingly, in the monotherapy group, patients with metastases to the liver, lung, adrenal glands, bone, or lymph nodes had a longer PFS than those without metastasis to these sites (Table 2 and Figure 2).

In our study, the monotherapy group included more patients with a high PD‐L1 expression than the combination therapy group. Although this imbalance could have affected the PFS and OS of the patients with a high PD‐L1 expression in the two study groups (Figure 1a,d), we did not observe a significant difference in the PFS and OS, even if patients did not have a high PD‐L1 expression (Figure 1c). Unlike previous studies, in the monotherapy group in this study there was no difference in PFS between the overall population and the population with a high PD‐L1 expression. This was because there was only one case with a PD‐L1 expression of 1–49%; this did not affect the overall population. In the phase III trials of pembrolizumab monotherapy among patients with PD‐L1 expression in at least 50% of the tumor cells, the median PFS was 309 days in the KEYNOTE‐024 study and 210 days in the KEYNOTE‐042 study.^4^, ^13^ Meanwhile, the median PFS of patients with a high PD‐L1 expression receiving combination therapy was 276 days in the KEYNOTE‐189 study and 240 days in the KEYNOTE‐407 study. These results suggest that the difference in the antitumor effects of monotherapy and combination therapy in patients with a high PD‐L1 expression is marginal. Given the lack of clinical trials that directly compare pembrolizumab monotherapy with combination therapy in patients with a high PD‐L1 expression, some researchers conducted a network meta‐analysis to compare the two regimens. Kim et al. reported that pembrolizumab in combination with chemotherapy achieved a slightly longer PFS than pembrolizumab monotherapy (HR 0.52, 95% CI 0.27–0.99), but did not improve the OS (HR 0.80, 95% CI 0.36–1.79).^14^ Dafni et al. also reported similar results (PFS: HR 0.52, 95% CI 0.38–0.77; OS: HR 0.73, 95% CI 0.50–1.05).^15^ Studies on atezolizumab, another ICI approved for the treatment of NSCLC, have also demonstrated little difference between atezolizumab monotherapy and combination therapy among patients with high PD‐L1 expression.^10^, ^11^, ^15^, ^16^, ^17^

Our results suggest that metastasis to the liver, lung, adrenal glands, bone, or lymph nodes is likely to be a predictive marker of longer PFS in patients treated with pembrolizumab monotherapy (Figure 2). This could be attributed to the differences in the tumor microenvironment of each metastatic lesion.^18^, ^19^ PD‐L1 expression on cancer cells in the metastatic sites differs from that on cancer cells in the primary tumor, thus possibly influencing the effect of ICIs. In advanced colorectal cancer, the expression of PD‐L1 in liver metastases is higher than that in the primary tumors.^20^ Hong et al. reported that PD‐L1 expression is highest in adrenal, liver, and lymph node metastases, and relatively lower in bone and brain metastases. Moreover, they also reported that a higher PD‐L1 expression in the lung or distant metastatic lesions was significantly associated with a better RR, PFS, and OS.^21^ Accumulation of anticancer immune cells at some metastatic sites might be another reason for better outcomes with ICIs. Ma et al. reported that PD‐1 expression on CD3^+^ T cells in tumors metastasizing to the lymph nodes is upregulated.^22^ Balatoni et al. revealed that invasion of the tumor microenvironment by immune cells, including CD4^+^ and CD8^+^ T cells and CD20^+^ B cells, was positively associated with the OS after treatment.^23^ One hypothesis is that monotherapy with ICIs promotes the antitumor effect of the immune cells at the site of metastasis and enhances the systemic immune reaction against cancer cells, thereby resulting in better outcomes. As chemotherapy can impair the antitumor effect of immune cells, the combination of ICI with chemotherapy could have a reduced efficacy in patients with metastasis (Figure 2b). Anestakis et al. reported that carboplatin treatment can increase the induction of bone marrow‐derived suppressor cells and deplete CD8^+^ T cells,^13^ suggesting that carboplatin may also suppress cellular immunity.^24^, ^25^, ^26^

There were some limitations to the current study. This was a retrospective, nonrandomized study, with a small sample size involving multiple institutions. This could have induced some biases that may have affected the generalizability of the findings. Moreover, the two study groups were substantially different, regarding the number of patients with a high PD‐L1 expression and the age of the patients. There were fewer patients with a high PD‐L1 expression in the combination therapy group; this may have resulted in no difference in PFS and OS (Figure 1a,c,d) in the overall population. Some metastasis‐specific prognostic factors, such as Royal Marsden Hospital scores and Gustave Roussy Immune scores in liver metastasis,^27^ and Tim‐4 positive cavity‐resident macrophages in malignant pleural effusion,^28^ were not analyzed in this study. Therefore, further studies with a larger sample size and prospective design are required to validate the findings from our study, especially in the area of immunological translational research.

In conclusion, pembrolizumab monotherapy and pembrolizumab in combination with chemotherapy do not demonstrate significant differences in the PFS, OS, and RR of patients with NSCLC; however, patients with NSCLC having metastases to specific sites could benefit more from pembrolizumab monotherapy rather than combination therapy.

## CONFLICT OF INTEREST

The authors have no conflicts of interest to declare.

## Supporting information


**Supporting information Figure S1** Kaplan–Meier analysis of progression‐free survival with and without metastasis to the liver, lung, bone, adrenal gland, or lymph nodes in the combination therapy group. Patients with metastasis to any of these sites had a shorter progression‐free survival than those who did not (*p* = 0.055, hazard ratio 2.50). PFS, progression‐free survival; CI, confidence interval
