# Supplementary figures and images for: Pembrolizumab monotherapy versus pembrolizumab plus chemotherapy in patients with non‐small‐cell lung cancer: A multicenter retrospective trial
Source: Thorac Cancer. 2021 Dec 5;13(2):228–35. doi: 10.1111/1759-7714.14252 (PMC8758435; doi:10.1111/1759-7714.14252)

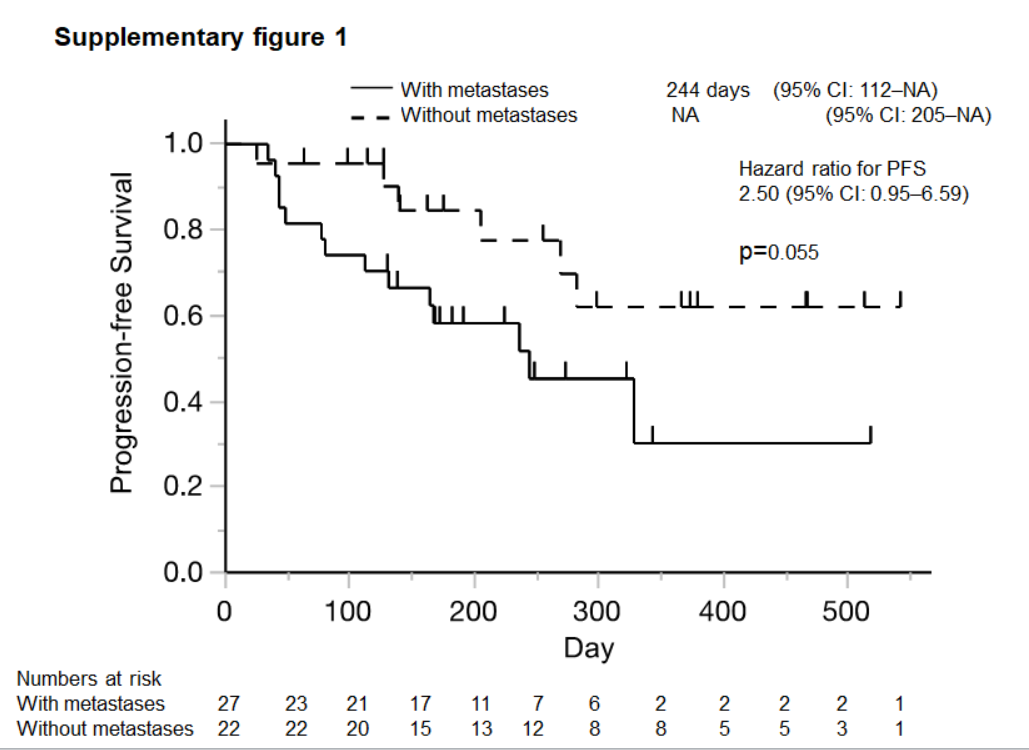

Supplement: Supplementary file 1 — Supporting information Figure S1 Kaplan–Meier analysis of progression‐free survival with and without metastasis to the liver, lung, bone, adrenal gland, or lymph nodes in the combination therapy group. Patients with metastasis to any of these sites had a shorter progression‐free survival than those who did not (p = 0.055, hazard ratio 2.50). PFS, progression‐free survival; CI, confidence interval [file TCA-13-228-s001.tif]
